# Genetic background- and environment-independent QTL and candidate gene identification of appearance quality in three MAGIC populations of rice

**DOI:** 10.3389/fpls.2022.1074106

**Published:** 2022-11-11

**Authors:** Huizhen Chen, Laiyuan Zhai, Kai Chen, Congcong Shen, Shuangbing Zhu, Pingping Qu, Jie Tang, Jianping Liu, Haohua He, Jianlong Xu

**Affiliations:** ^1^ Key Laboratory of Crop Physiology, Ecology and Genetic Breeding, Ministry of Education/College of Agronomy, Jiangxi Agricultural University, Nanchang, Jiangxi, China; ^2^ Shenzhen Branch, Guangdong Laboratory for Lingnan Modern Agriculture, Agricultural Genomics Institute at Shenzhen, Chinese Academy of Agricultural Sciences, Shenzhen, China; ^3^ Pingxiang Center for Agricultural Sciences and Technology Research, Pingxiang, Jiangxi, China; ^4^ The National Key Facility for Crop Gene Resources and Genetic Improvement, Institute of Crop Sciences, Chinese Academy of Agricultural Sciences, Beijing, China

**Keywords:** grain appearance quality, genome-wide association study (GWAS), linkage analysis (LA), quantitative trait locus/loci (QTL), designed QTL pyramiding

## Abstract

Many QTL have been identified for grain appearance quality by linkage analysis (LA) in bi-parental mapping populations and by genome-wide association study (GWAS) in natural populations in rice. However, few of the well characterized genes/QTL have been successfully applied in molecular rice breeding due to genetic background (GB) and environment effects on QTL expression and deficiency of favorable alleles. In this study, GWAS and LA were performed to identify QTL for five grain appearance quality-related traits using three multi-parent advanced generation inter-cross (MAGIC) populations. A total of 22 QTL on chromosomes 1-3, 5-8 were identified by GWAS for five traits in DC1, DC2 and 8way, and four combined populations DC12 (DC1+DC2), DC18 (DC1+8way), DC28 (DC2+8way) and DC128 (DC1+DC2+8way). And a total of 42 QTL were identified on all 12 chromosomes except 10 by LA in the three single populations. Among 20 QTL identified by GWAS in DC1, DC2 and 8way, 10, four and three QTL were commonly detected in DC18, DC28, and DC128, respectively. Similarly, among 42 QTL detected by LA in the three populations, four, one and two QTL were commonly detected in DC18, DC28, and DC128, respectively. There was no QTL mapped together in DC12 by both two mapping methods, indicating that GB could greatly affect the mapping results, and it was easier to map the common QTL among populations with similar GB. The 8way population was more powerful for QTL mapping than the DC1, DC2 and various combined populations. Compared with GWAS, LA can not only identify large-effect QTL, but also identify minor-effect ones. Among 11 QTL simultaneously detected by the two methods in different GBs and environments, eight QTL corresponded to known genes, including *AqGL3b* and *AqGLWR3a* for GL and GLWR, *AqGW5a*, *AqGLWR5*, *AqDEC5* and *AqPGWC5* for GW, GLWR, DEC and PGWC, and *AqDEC6b* and *AqPGWC6b* for DEC and PGWC, respectively. *AqGL7*, *AqGL3c*/*AqGLWR3b*, *AqDEC6a*/*AqPGWC6a*, and *AqPGWC7* were newly identified and their candidate genes were analyzed and inferred. It was discussed to further improve grain appearance quality through designed QTL pyramiding strategy based on the stable QTL identified in the MAGIC populations.

## Introduction

Rice (*Oryza sativa*) is one of the most important cereal crops grown worldwide, feeding more than half of the world population. With the everlasting growth of population and development of the economy and living standards, high yield with good grain quality has becomes the prime objective of breeding (Wang et al., 2017; Murchie et al., 2009). Among grain quality, appearance quality is responsible for final rice market value. Grain appearance quality are mainly associated with grain shape, which is specified by grain length (GL), grain width (GW), the ratio of grain length to width (GLWR) and grain thickness. The slender grains with grain length-to-width ratio larger than 3 are preferred by most rice consumers from Southern China, the USA, and South and Southeast Asian countries (Gong et al., 2017). As most important component of appearance quality, chalkiness is usually evaluated by the degree of endosperm chalkiness (DEC) and the percentage of grain with chalkiness (PGWC). DEC and PGWC are easily affected by growing environment (Zhao et al., 2015; You et al., 2022).

To date, a lot of QTL affecting appearance quality of rice have been identified through linkage mapping and association study (Zhao et al., 2011; Singh et al., 2012; Zhang et al., 2013; Yang et al., 2014; Liu et al., 2015a; Qiu et al., 2015; Wang et al., 2017). Moreover, many genes associated with grain shape have been identified and cloned by linkage mapping and association study, such as *GS2* (Hu et al., 2015), *GS3* (Mao et al., 2010), *GS5* (Li et al., 2011), *GS6* (Sun et al., 2013), *GS9* (Zhao et al., 2018), *GW2* (Song et al., 2007), *GW5* (Weng et al., 2008; Liu et al., 2017), *GW8* (Wang et al., 2012), *GW3* and *GW6* (Guo et al., 2009), *GW6a* (Song et al., 2015), *GIF1* (Wang et al., 2008), *qSW5* (Shomura et al., 2008), *qGL3* (Zhang et al., 2012), *GL3.1* (Qi et al., 2012), *GL7/GW7* (Wang et al., 2015a; Wang et al., 2015b), *OsMAPK6* (Liu et al., 2015a), *GLW7* (Si et al., 2016), and *BG1* (Liu et al., 2015b). These cloned genes have provided considerable insight into the individual molecular basis of grain size and shape regulation. For grain chalkiness, only one gene, *Chalk5* had been cloned (Li et al., 2014). One QTL, *qPGWC-7* for *PGWC* was fine mapped to 44 kb region on chromosome 7 (Zhou et al., 2009), and one QTL cluster for chalkiness flanked by id4007289 and RM252 on chromosome 4 was detected by single environment analysis and joint mapping across nine environments (Zhao et al., 2016).

Genome-wide association analysis and linkage analysis (LA) are two mainstream methods for QTL mapping for complex quantitative traits. At present, most genes were cloned by linkage analysis, i.e., forward genetic cloning approach, which had the advantage of high localization accuracy. In addition, genetic populations used for LA were generally constructed by repeated hybridization and self-crossing of two or more materials. Therefore, LA involved only two or several alleles at the same locus, and parental material could only represent a small portion of the phenotypic variation associated with the species. With the development of sequencing technology, some genes related to grain weight and grain shape in rice, were excavated by genome-wide association study (GWAS) using natural germplasm populations, such as *GW5* (Liu et al., 2017), *OsLG3* (Yu et al., 2017) and *GLW7* (Si et al., 2016). The natural germplasm populations used for association analysis were generally with extensive variation and there was no need to construct genetic segregation populations. It could simultaneously identify multiple alleles at the same locus and then applied the superior alleles directly to breeding practice. However, it was difficult to map the rare alleles as for that SNP of rare alleles were normally filtered out during GWAS.

Many QTL have been reported for yield and grain quality traits using various bi-parental and association mapping populations. However, few of the well characterized genes/QTL have been successfully used in breeding for enhanced trait performance. The main reasons are that the mapping populations used are generally separate from breeding populations, and the favourable alleles for molecular breeding can’t be easily mined in the population derived from bi-parents. Although association mapping using diversity panels can identify favourable alleles, it is difficult to directly be exploited in breeding because most of the accessions had poor performance in many important agronomic traits. As a matter of fact, multi-parent mapping populations such as multi-parent advanced generation inter-cross (MAGIC) (Cavanagh et al., 2008) and nested association mapping (NAM) (Yu et al., 2006) populations have been developed for many crops, which are considered as an ideal integration of QTL mapping and practical breeding. In view of the complementary advantages of association analysis and linkage analysis, mapping results were more reliable as for the positioning results of the two methods can be compared and verified each other. In this study, we performed LA and GWAS to detect QTL related to rice appearance quality using MAGIC populations developed by eight parents, to compare genetic background (GB) effect on QTL detection and efficacy of the two mapping approaches, and identify candidate genes for identified novel QTL. Our results will enhance the knowledge of the genetic basis of appearance quality and provide valuable information for improving appearance quality in rice breeding program.

## Materials and methods

### Plant materials

Eight elite *indica* (*xian*) parents, were selected based on genetic diversity to develop the three multi-parental RIL populations at the IRRI since 2008 (Meng et al., 2016). Four single crosses were made from the eight homozygous parents, denoted (A × B), (C × D), (E × F) and (G × H). Two four-way crosses were then generated from the four single crosses F_1_ plants, denoted (A × B) × (C × D) and (E × F) × (G × H), and advanced separately by single-seed descent (SSD) to produce two four-parental RIL populations (denoted DC1 and DC2). An eight-way cross was made by intercrossing 100 F_1_ plants of the four-way cross ABCD and 100 F_1_ plants of the four-way cross EFGH, denoted [(A × B) × (C × D)] × [(E × F) × (G × H)]. The eight-parental RIL population was produced by SSD from 1000 eight-way cross F_1_ plants (denoted 8way). Three multiple-parental RIL populations, including 368 lines of DC1, 374 lines of DC2 and 561 lines of 8way, were used for materials for trait investigation.

### Field trial and phenotypic investigation

Field trials were conducted at Pingxiang (PX) (113.85°N, 27.6°E) of Jiangxi province and Shenzhen (SZ) (22.6°E, 114.07°N) of Guangdong province both in 2015 and 2016. The three RIL populations were planted in a randomized complete block design with two replications and 10 plants in a row for each replication. The plant spacing was 17 cm between individual plants and 20 cm within each row.

At maturity, eight uniform plants in the middle of each plot were bulk-harvested and air-dried for three months in the drying houses. Then, around 150 g seeds were dehulled in an electrical dehuller (model JLGJ45, China) and milled by a desk-top rice miller (JNMJ 6, China). All full head milled rice kernels of each accession were used to measure grain length (GL, mm), grain width (GW, mm), grain length-width ratio (GLWR), degree of endosperm chalkiness (DEC, %), percentage of grain with chalkiness (PGWC, %) using a rice grain appearance quality scanning machine (SC-E, Wanshen Technology Company, Hangzhou, China). The average trait value of each accession was used in data analyses (**Supplementary Table S1**).

### Genotyping

Genotyping of the three MAGIC populations were reported by Meng et al. (2017). Specifically, genomic DNA for SNP genotyping was isolated from fresh leaf samples of 5-week-old seedlings using a modified cetyltrimethylammonium bromide (CTAB) method (Murray and Thompson, 1980). Genotyping was performed using a customized rice 56 K SNP array containing 56,897 SNP screened from the 3 K Rice Genome Project (3K RGP, 2014). Those SNPs with missing rate over 20% and minor allele frequency (MAF) less than 5% were removed. Finally, 28529 high-quality SNPs were selected and used for genetic diversity analysis, GWAS and linkage analysis (LA) for the three populations in this study.

### Analysis of genetic diversity

A total of 28529 SNP markers were used to assess the genetic diversity of the DC1, DC2, 8-way, DC1_parents, DC2_parents, and 8way_parents by estimating minor allele frequency (MAF), expected heterozygosity (He), and polymorphism information content (PIC) using plink V1.90 (Purcell et al., 2007).

### Genome-wide association study

We performed a GWAS to identify the SNPs significantly associated with all measured traits of the three single-populations (DC1, DC2 and 8way), using the selected SNPs and the mean trait values of the accessions. In order to analyze effect of genetic background (GB) on QTL mapping by GWAS and further to improve resolution of QTL mapping, we combined different single-population, including DC12 (DC1+DC2), DC18 (DC1+8way), DC28 (DC2+8way) and DC128 (DC1+DC2+8way) to expand the population size and performed GWAS using the selected SNPs and the mean trait values. In this study, the model of mixed linear (MLM), PCA + K, was used in the association analysis. And we performed GWAS by the software GAPIT (Lipka et al., 2012). After GWAS, the important SNPs affecting the measured traits were claimed when the test statistics reached *P <*1.0 × 10^−4^ in at least two environments or two traits.

### Genetic linkage analysis

The linkage maps of three populations were constructed by GAPL V1.2 (Qu et al., 2020). QTL mapping of the five traits were conducted separately in DC1, DC2 and 8way by using ICIM with the PLQ function in GAPL V1.2 (Zhang et al., 2019), the scanning step was set at 0.1 cM. Probabilities of adding and removing variables in stepwise regression were set at 0.001 and 0.002, respectively. A 1000-permutation test was performed to establish a LOD threshold of 3.0 at a 95% confidence level.

### Candidate gene identification for the important QTL

Based on the mapping results of GWAS and LA, the QTL simultaneously detected by the two methods were used as important QTL for searching candidate gene affecting the measured traits. Besides, stable QTL, identified at least two environment or two populations by GWAS or LA, were also used as important QTL for candidate gene analysis.

Gene-based association analysis was carried out to detect candidate genes for important QTL. The following steps were conducted to identify candidate genes for important QTL identified. We, firstly, found the SNP inside genes which located in the region of 100 kb each side of the peak SNP. The genotype manipulation was done in the same way as described above. Then, the high-quality SNPs inside of these regions were used to perform gene-based association analysis through MLM using the PCA and K. Thirdly, SNPs associated with a *P*-value of <0.05 were considered significant, and the genes in which the SNP was located was inferred as candidate genes affecting the measured traits. Fourthly, haplotype analysis was carried out in 731 accessions selected from the 3K (3K RGP, 2014) with grain quality data collected in Shenzhen (SZ) and Sanya (SY) in 2015 for each of the candidate genes in each important QTL region using all non-synonymous SNPs located inside of the gene CDS region. Finally, candidate genes were determined by testing the significant differences among major haplotypes (containing more than 10 samples) for each important QTL through ANOVA.

## Results

### Genetic diversity

The results of genetic diversity were summarized in **Table 1** for the three populations (DC1, DC2 and 8way) and their parents. The average MAF of the three MAGIC populations were all greater than 0.1, suggesting the MAGIC populations had much better balance in allele frequencies. As expected, there were more polymorphic markers in the 8way population than in DC1 and DC2. The He of the 8way population was higher than the other two populations. The DC1 and DC2 had similar He and PIC, which were slightly lower than those in 8way population. The average He and PIC of the DC1 and DC2 were very similar to those of their parent populations, and those of the 8way were larger than those of 8way_parents, indicating the good quality of these three MAGIC populations.

**Table 1:** Genetic diversity statistics of the DC1, DC2, 8way and their parental line populations.

Population	Sample	MAF ^a^	PIC ^b^	He ^c^
DC1	368	0.2522	0.2667	0.3342
DC2	374	0.2333	0.2505	0.3124
8way	561	0.2509	0.2764	0.3436
DC1_parents	4	0.2528	0.2534	0.3209
DC2_parents	4	0.2533	0.2585	0.3269
8way_parents	8	0.2103	0.2036	0.2593

a minor allele frequency.

b polymorphism information content.

c expected heterozygosity.

### Phenotypic variations and correlations

**Figure 1:**
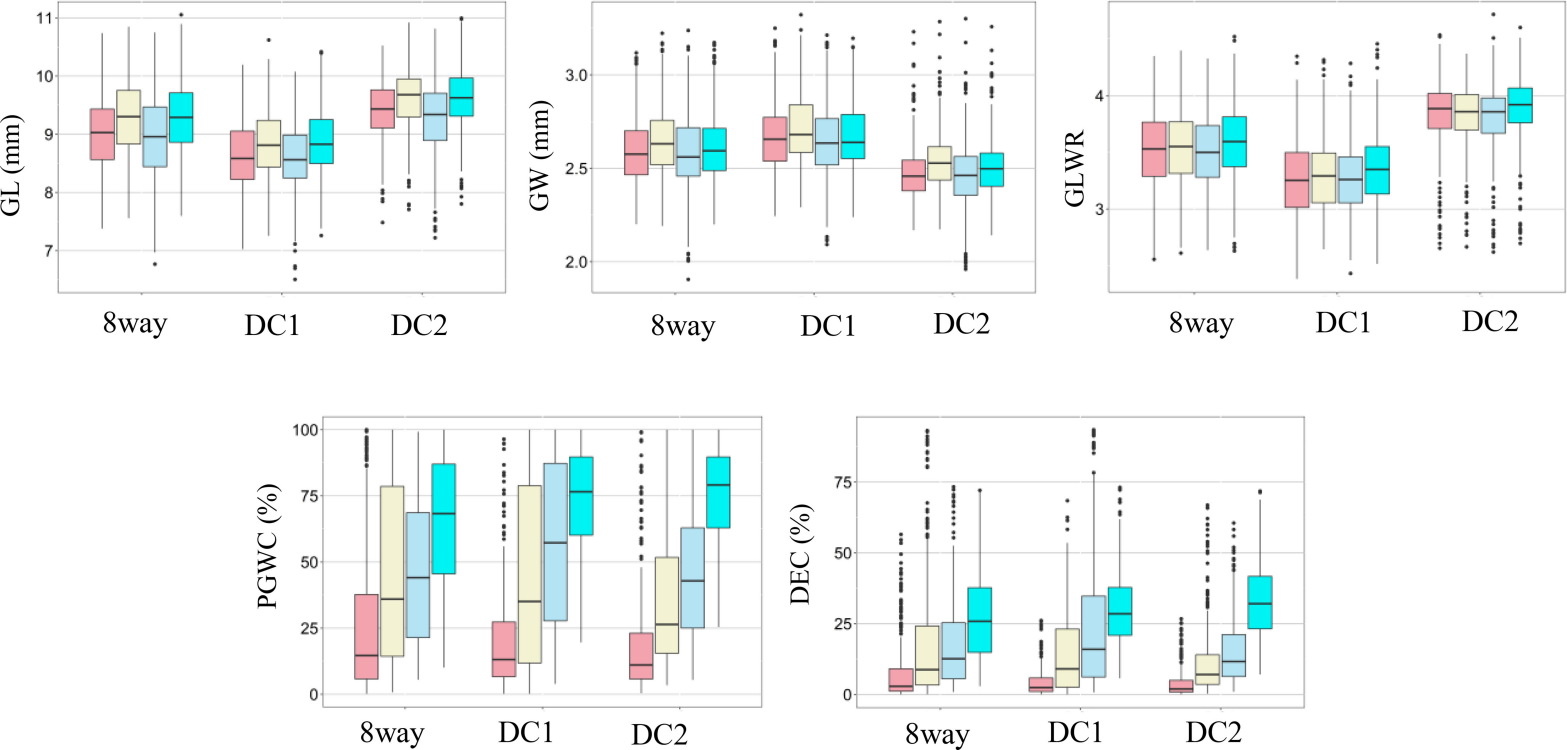
Box plots of five rice grain shape and appearance quality traits in two environments and years. GL, Grain length; GW, Grain width; GLWR, Grain length to width ratio; DEC, Degree of endosperm chalkiness; PGWC, Percentage of grains with chalkiness. Light pink, light yellow, light blue and cyan colors indicate 2015 in PX, 2015 in SZ, 2016 in PX and 2016 in SZ, respectively.

The rice panel used in this study showed wide variations for all the measured traits and most traits appeared to be normally distributed (**Figure 1**). Significant variations among different environments were observed for DEC and PGWC, but not for GL, GW and GLWR, and the three multi-parental RIL populations showed consistent performances (**Figure 1**), indicating DEC and PGWC were easily influenced by environment. Among them, three lines, L1352, L1422 and L1692 were selected with largest GLWR (4.18) and had higher average appearance quality with the PGWC (DEC) of 14.21% (3.58%), 17.33% (5.43%), and 11.74% (4.74%) in four environments respectively. Besides, another three lines, L1660, L1716 and L1745 were selected with lowest PGWC (<10%) and DEC (<3%) and relatively large averaged GLWR with 3.65, 3.54 and 3.96 in four environments, respectively (**Supplementary Table S2**).

**Figure 2:**
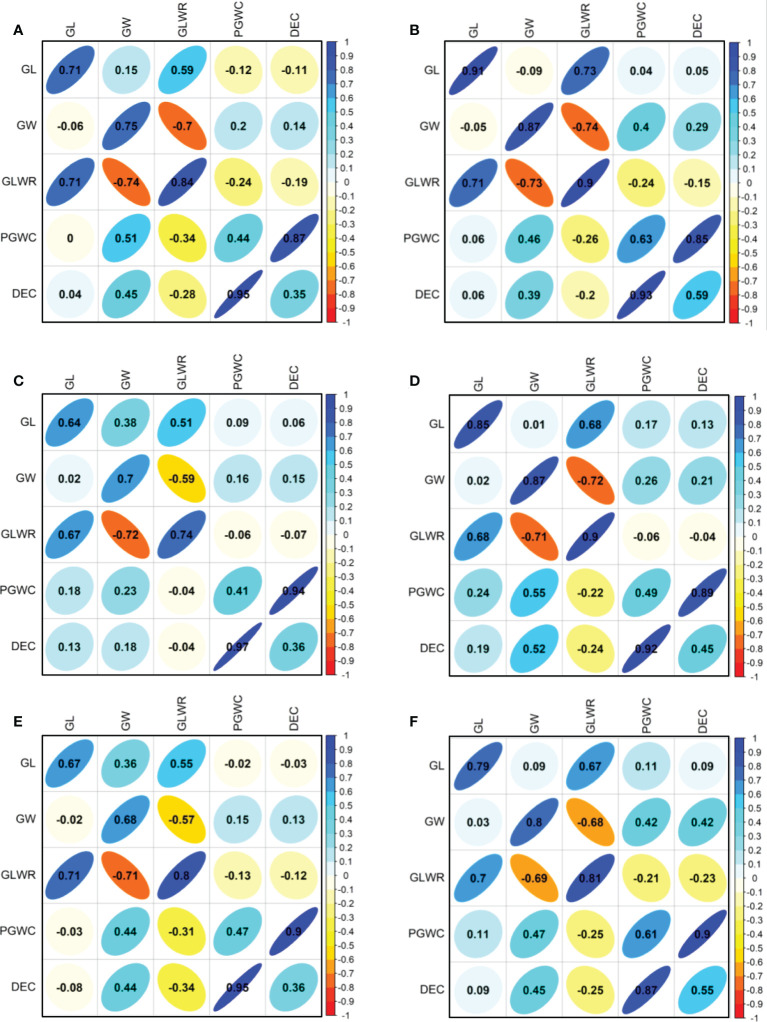
Correlations between five evaluated traits in 2015 (lower triangular) and 2016 (upper triangular). **(A, B)** Represent DC1 population in PX and SZ, respectively; **(C, D)** Represent DC2 population in PX and SZ, respectively; **(E, F)** Represent 8way population in PX and SZ, respectively. The values on principal diagonal indicated correlations between 2015 and 2016. The values were correlation coefficients (r). The areas and colors of ellipses showed the absolute value of corresponding r. Right and left oblique ellipses indicated positive and negative correlations, respectively.

The phenotype pairwise correlations between the measured traits were similar in all four environments and three populations (**Figure 2**). As expected, GLWR showed significantly positive correlations with GL, but negative correlations with GW. Significantly positive correlations were observed between DEC and PGWC. GW showed significantly positive correlations with PGWC and DEC in all three populations in SZ. While in PX, GW only showed positive correlations with PGWC and DEC in DC1 and 8way populations in 2015. There were strong positive correlations between 2015 and 2016 for all measured traits in the three populations.

### QTL detection by GWAS

#### Basic statistics of markers

Total of 28529 SNPs was used in each of the three RIL populations (DC1, DC2 and 8way) in this study. The whole-genome size was 374.82 Mb with the average spacing 13.14 kb between two adjacent SNPs in each population. The size of chromosome varied from 22.69 Mb for chromosome 9 to 43.95 Mb for chromosome 1 and the average marker spacing ranged from 11.36 kb for chromosome 1 to 16.37 kb for chromosome 12 (**Table 2**).

**Table 2:** Distributions of markers used in GWAS on chromosomes.

Chr	Start (bp) ^a^	End (bp) ^b^	Size (Mb)	Count	Average spacing (kb)
1	80943	43954572	43.95	3870	11.36
2	3546	35930927	35.93	2922	12.30
3	180076	36413228	36.41	2659	13.69
4	59046	36474610	36.47	2948	12.37
5	7169	29831609	29.83	2260	13.20
6	123035	30968378	30.97	2417	12.81
7	17741	29671234	29.67	2339	12.68
8	17010	28438367	28.44	1959	14.52
9	44837	22692165	22.69	1788	12.69
10	46485	23066742	23.07	1636	14.10
11	123694	29876538	29.88	2050	14.58
12	105460	27514251	27.51	1681	16.37
Total			374.82	28529	13.14

a The starting physical position of the SNP used in GWAS on chromosomes.

b The ending physical position of the SNP used in GWAS on chromosomes.

#### Population structure

**Figure 3:**
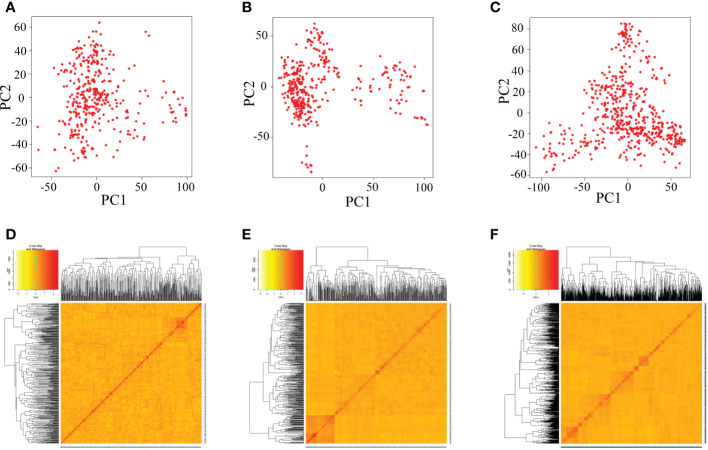
Analysis of the population structure of three populations by GAPIT. **(A–C)** Indicate the PCA plots of DC1, DC2 and 8way, respectively; **(D–F)** indicate the kinship matrix of DC1, DC2 and 8way, respectively.

There was no obvious clustering based on the principal component analysis and kinship analysis, suggesting that only one group or no obvious population structure in the all three populations (**Figure 3**), which might be due to random recombination in the segregation generations during establishment of population.

### QTL identified by GWAS

A total of 22 QTL for five traits were identified in the three single populations (DC1, DC2 and 8way) and four combined populations (DC12, DC18, DC28 and DC128), ranging from three QTL for GLWR to six QTL for DEC (**Supplementary Table S3**). Among them, 15 QTL were detected in at least two populations and three QTL (*AqGL3b*, *AqGLWR3a*, *AqPGWC6b*) were detected in the all seven populations.

For GL, four QTL were detected on chromosomes 3 and 7 (**Supplementary Table S3**). Among them, *AqGL3a* was only detected on chromosomes 3 in single population DC2. *AqGL3b* was detected on chromosomes 3 in the three single populations (DC1, DC2 and 8way) and four combined populations (DC12, DC18, DC28 and DC128). *AqGL3c* was detected on chromosomes 3 in all populations except for DC2. *AqGL7* was mapped on chromosomes 7 in populations DC2, DC28 and DC128.

Four QTL for GW were detected on chromosomes 1, 2, and 5 (**Supplementary Table S3**). *AqGW1* and *AqGW2* were detected in 8way and DC128, respectively. *AqGW5a* was detected on chromosome 5 in the all populations except for DC2. *AqGW5b* was detected on chromosome 5 in DC2 and 8way.

Three QTL for GLWR were detected on chromosomes 3 and 5 (**Supplementary Table S3**). *AqGLWR3a* was detected on chromosome 3 in the all seven populations. *AqGLWR3b* was detected on chromosome 3 in the all populations except for DC2 and DC12. *AqGLWR5* was detected on chromosome 5 in the all populations except for DC2.

For DEC, a total of six QTL were detected on chromosomes 2, 5, 6 and 8 (**Supplementary Table S3**). Among them, *AqDEC2*, *AqDEC6c* and *AqDEC8* were only detected in DC1, DC1 and DC2, respectively. *AqDEC5* was detected on chromosomes 5 in the all populations except for DC2. *AqDEC6a* was detected on chromosomes 6 in populations 8way, DC12 and DC28. *AqDEC6b* was detected on chromosomes 6 in all populations except for DC2.

For PGWC, five QTL were mapped on chromosomes 5, 6, 7 and 8 (**Supplementary Table S3**). Among them, *AqPGWC8* was only detected in DC2. *AqPGWC5* was detected on chromosome 5 in all populations except for DC2. *AqPGWC6a* and *AqPGWC7* were detected in 8way and DC28, and DC28 and DC128, respectively. *AqPGWC6b* was detected on chromosomes 6 in all three single and four combined populations.

### QTL analysis by genetic linkage maps

In order to compare QTL mapping by GWAS, QTL mapping of the five traits were conducted separately in DC1, DC2 and 8way by using ICIM with the PLQ function in GAPL V1.2. A total of 42 QTL were detected in the three populations, including 16, 19 and 16 QTL in DC1, DC2 and 8way, respectively (**Supplementary Table S4**).

A total of 16 QTL for the five traits were detected on chromosomes 1, 3, 4, 5, 6, 8, 9 and 11 in DC1 in four environments, including four QTL for GL, two for GW, five for GLWR, two for DEC and three for PGWC (**Supplementary Table S4**). Among them, *GqGL3a* and *GqGLWR3b* were mapped together on chromosome 3 in all four environments, and explained the average phenotypic variances of 19.66% and 10.07%, respectively. *GqGW5a*, *GqGLWR5*, *GqDEC5a*, and *GqPGWC5a* were identified in the same region on chromosome 5 at least three environments, and explained the average phenotypic variances of 34.07%, 21.06%, 6.70%, and 11.87%, respectively. *GqDEC6* and *GqPGWC6* were mapped together on chromosome 6 with average PVE of 7.14% and 15.95%, respectively. *GqGL1a* and *GqGLWR9* were detected for GL and GLWR under three environments, and averagely accounted for phenotypic variances of 4.29% and 4.03%, respectively.

A total of 19 QTL were detected in DC2 on all chromosomes except 5, 6 and 10, including three for GW and four for each of GW, GLWR, DEC and PGWC (**Supplementary Table S4**). *GqGL3a*, *GqGW2b*, *GqGLWR3b* and *GqPGWC1a* were detected in all four environments with the average phenotypic variances of 10.00%, 5.43%, 6.00%, and 4.23%, respectively. Moreover, *GqGL3a* and *GqGLWR3b* were simultaneously identified on chromosome 3. *GqGL2a*, *GqGLWR4* and *GqDEC12* were detected in three environments with the average phenotypic variances of 7.48%, 5.12%, and 3.95%, respectively. Moreover, *GqGW8* and *GqGLWR8* were mapped together on chromosome 8 in three environments with average phenotypic variances of 6.59% and 11.70%, respectively.

In 8way, a total of 16 QTL were identified on chromosomes from 1 to 6, including three for GL, one for GW, five for GLWR, three for DEC and four for PGWC (**Supplementary Table S4**). Specifically, *GqGL3a* was only identified for GL in all four environments with average phenotypic variance of 6.00%. *GqGL3b* and *GqGLWR3b* were identified for GL and GLWR in three environments with average phenotypic variances of 6.93% and 4.43%, respectively. In addition, *GqGL1b* and *GqGLWR1*, *GqGL3b* and *GqGLWR3c*, *GqGW5* and *GqGLWR5*, *GqDEC5b* and *GqPGWC5c*, *GqDEC5c* and *GqPGWC5b*, and *GqDEC6* and *GqPGWC6* were mapped together on chromosomes 1, 3, 5, 5, 5, 6, respectively.

### Effect of GB and population size on QTL mapping

Among 20 QTL identified for five traits by GWAS in DC1, DC2 and 8way, 10 QTL (*AqGL3b*, *AqGLWR3a*, *AqPGWC6b, AqDEC5*, *AqDEC6b*, *AqGL3c*, *AqGLWR3b*, *AqGLWR5*, *AqGW5a* and *AqPGWC5*) were detected both in DC1 and 8way, four QTL (*AqGL3b*, *AqGLWR3a*, *AqPGWC6b* and *AqGW5b*) were detected both in DC2 and 8way, and three QTL (*AqGL3b*, *AqGLWR3a* and *AqPGWC6b*) were detected in all the three single populations (DC1, DC2 and 8way), but no QTL was mapped together in DC1 and DC2. These mainly resulted from different GB in different populations. The GB of DC1 and DC2 was partially the same as that of 8way, while that of DC1 and DC2 were completely different. These results indicated that GB could affect the mapping results, and it was easier to map the common QTL in similar GB.

Among 42 QTL detected for the five traits by LA in the three populations, *GqGL3a* and *GqGLWR3b* were detected in DC1, DC2 and 8way, four QTL (*GqDEC6*, *GqGLWR5*, *GqGW5a* and *GqPGWC6*) were detected both in DC1 and 8way, and *GqGLWR4* was only detected in both DC2 and 8way. There was no QTL simultaneously detected both in DC1 and DC2. These results confirmed that GB could affect the mapping results as indicated by GWAS.

In order to further reveal the effect of population size on mapping results, we combined different single populations to expand the population size and performed GWAS for the five traits using the same set of SNPs and the mean trait values. A total of 10, 10, 14, and 13 QTL were identified in DC12, DC18, DC28 and DC128, respectively. The number of QTL mapped in the single population was comparable to those mapped in the merged populations. And the QTL intervals were similar for those QTL commonly detected in single and combined populations. It was indicated that GWAS can’t obviously improve mapping results using the merged populations which were derived from relative larger single cross populations.

### Candidate gene analysis of important QTL

QTL simultaneously detected by GWAS and LA, including *AqGL3b*, *AqGL3c*, *AqGL7, AqGW5a, AqGLWR3a, AqGLWR3b, AqGLWR5, AqDEC5, AqPGWC5, AqDEC6b*, and *AqPGWC6b* could be used as important QTL for excavating candidate genes associated with rice appearance quality. Besides, *AqDEC6a, AqPGWC6a* and *AqPGWC7* stably detected in different environments and populations by GWAS were also included in candidate gene analysis. Among the important QTL, *AqGL3b* and *AqGLWR3a* were coincided with cloned gene *GS3* for grain size in rice (Mao et al., 2010). The QTL cluster (*AqGW5a, AqGLWR5, AqDEC5* and *AqPGWC5*) was coincided with the cloned gene *GW5* (Weng et al., 2008). Two QTL (*AqDEC6b* and *AqPGWC6b*) were coincided with the cloned gene *GS6* (Sun et al., 2013). So, *AqGL3c*/*AqGLWR3b, AqDEC6a/AqPGWC6a*, *AqGL7*, and *AqPGWC7* were considered as newly identified QTL for rice appearance quality in this study, and would be used for further candidate gene analysis and favourable gene mining.

**Figure 4:**
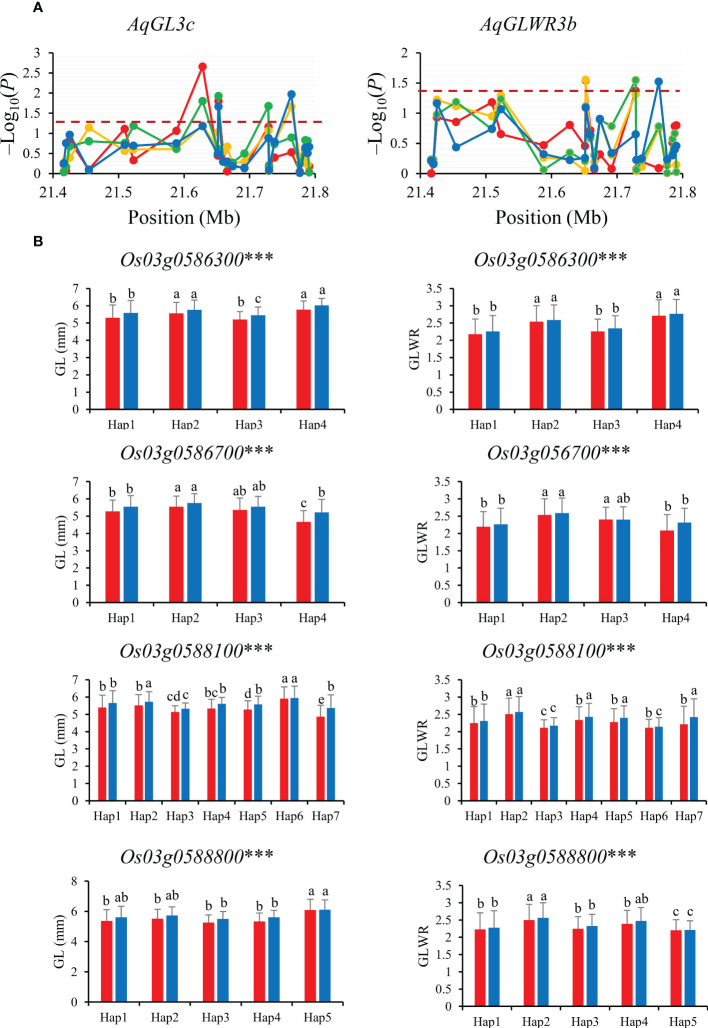
Gene-based association analysis **(A)** and haplotypes analysis of candidate genes **(B)** of *AqGL3c/AqGLWR3b.* Dash line showed the threshold to determine significant SNP. The *** suggested significance of ANOVA at *p* < 0.001. The letter on histogram (A–E) indicated multiple comparisons result at the significant level 0.05. Red, orange, green and blue color of lines indicated 15_PX, 16_PX, 15_SZ and 16_SZ environments, respectively. The red and blue color of the histogram indicated SY and SZ in 2015, respectively.

Gene-based association analysis of *AqGL3c*/*AqGLWR3b* for two traits (GL and GLWR) were conducted in combined-population DC128 through MLM using the PCA and K. A total of four significant SNPs was identified within the region from 21.35 Mb to 21.80 Mb on chromosome 3, and these significant SNPs fell on four genes (*Os03g0586300*, *Os03g0586700*, *Os03g0588100* and *Os03g0588800*) (**Figure 4**). Haplotype analysis indicated that all the four genes showed significant differences in GL and GLWR among haplotypes in 3K, suggesting the four genes could be candidate genes for GL and GLWR.

**Figure 5:**
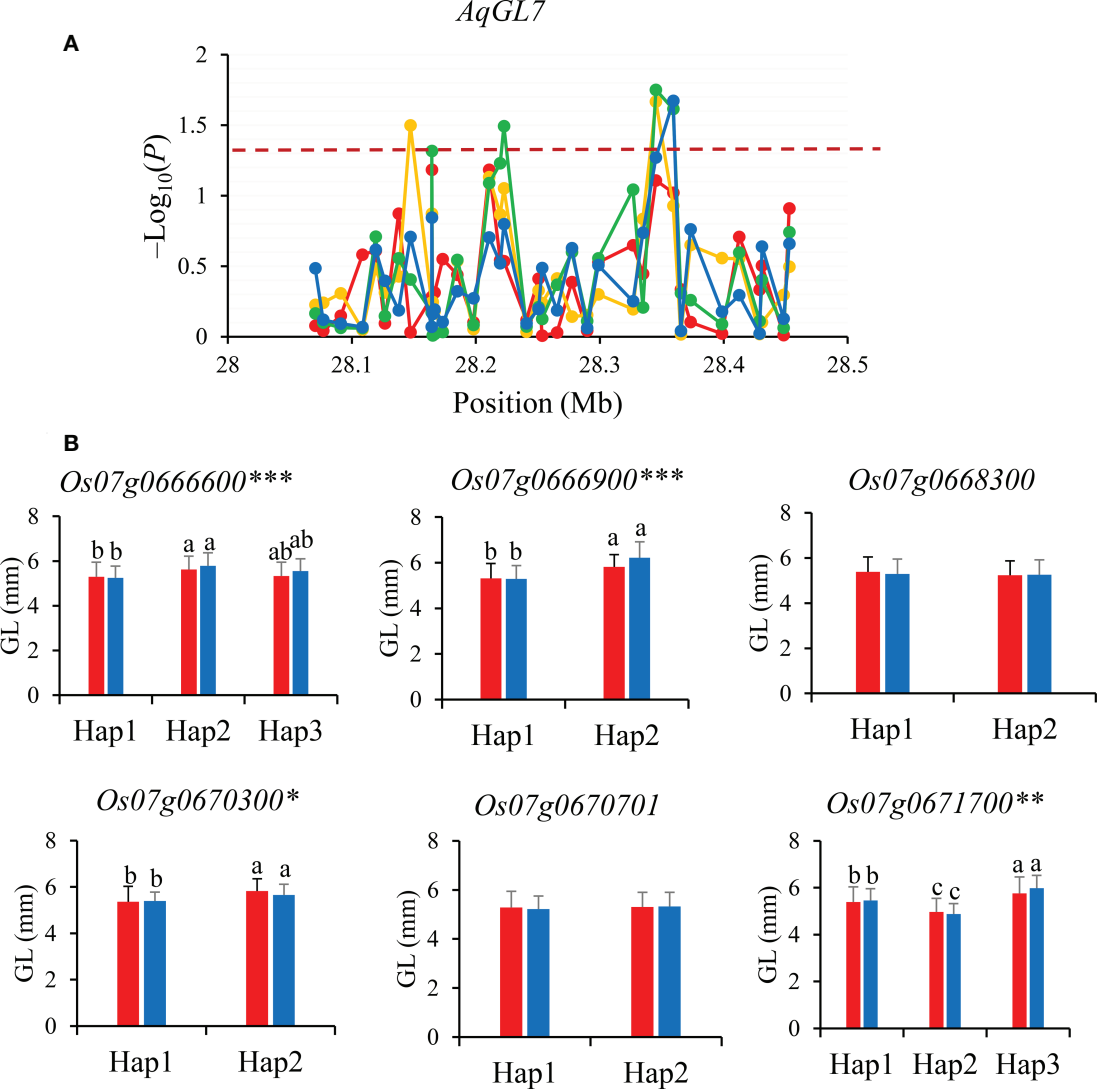
Gene-based association analysis **(A)** and haplotypes analysis of candidate genes **(B)** of *AqGL7*. Dash line showed the threshold to determine significant SNP. The *, ** and *** suggested significance of ANOVA at *p* < 0.05, *p* < 0.01 and *p* < 0.001, respectively. The letter on histogram (a, b and c) indicated multiple comparisons result at the significant level 0.05. Red, orange, green and blue color of lines indicated 15_PX, 16_PX, 15_SZ and 16_SZ environments, respectively. The red and blue color of the histogram indicated SY and SZ in 2015, respectively.

Similarly, gene-based association analysis of *AqGL7* for GL was conducted in combined-population DC128 through MLM using the PCA and K. Total of seven significant SNPs were tested and six candidate genes (*Os07g0666600*, *Os07g0666900*, *Os07g0668300*, *Os07g0670300*, *Os07g0670701* and *Os07g0671700*) were identified based on the significant SNPs (**Figure 5**). Of them, only four genes (*Os07g0666600*, *Os07g0666900*, *Os07g0670300*, and *Os07g0671700*) showed significant differences in GL among different haplotypes in 3K, and were inferred as candidate genes for GL.

**Figure 6:**
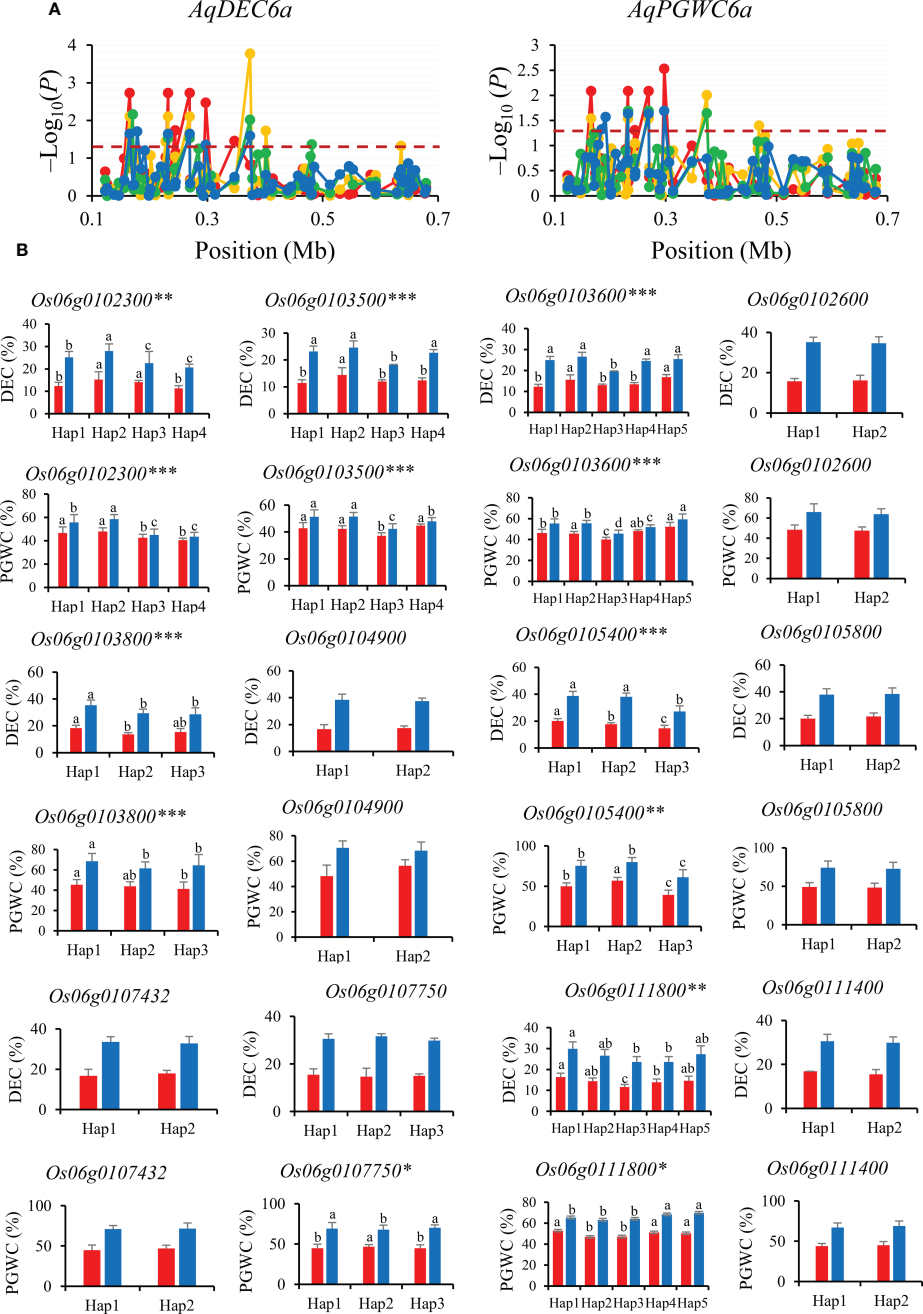
Gene-based association analysis **(A)** and haplotypes analysis of candidate genes **(B)** of *AqDEC6a/AqPGWC6a.* Dash line showed the threshold to determine significant SNP. The *, ** and *** suggested significance of ANOVA at *p* < 0.05, *p* < 0.01 and *p* < 0.001, respectively. The letter on histogram (A–D) indicated multiple comparisons result at the significant level 0.05. Red, orange, green and blue color of lines indicated 15_PX, 16_PX, 15_SZ and 16_SZ environments, respectively. The red and blue color of the histogram indicated SY and SZ in 2015, respectively.

*AqDEC6a* and *AqPGWC6a* were mapped in 0−0.68 Mb for DEC and PGWC on chromosomes 6. A total of nine significant SNPs for DEC and seven significant SNPs for PGWC were identified within this region and four of them were significantly associated with both DEC and PGWC (**Figure 6A**). Then, the 12 genes (*Os06g0102300, Os06g0103500, Os06g0103800, Os06g0104900, Os06g0105400, Os06g0105800, Os06g0107750, Os06g0111400, Os06g0111800, Os06g0102600, Os06g0103600* and *Os06g0107432*) were performed haplotype analysis to detected the candidate genes affecting DEC and PGWC in 8way. Among them, six genes (*Os06g0102300, Os06g0103500, Os06g0103800, Os06g0105400, Os06g0111800* and *Os06g0103600*) showed significant differences in both DEC and PGWC among different haplotypes in 3K, thus they could be inferred as candidate genes for DEC and PGWC (**Figure 6B**).

For *AqPGWC7*, however, there was no differences among different haplotypes for PGWC through gene-based association and haplotype analyses.

## Discussion

### Phenotypic variation of appearance quality of rice in MAGIC populations

In general, grain appearance was mainly determined by grain shape (GL, GW and GLWR) and chalkiness (DEC and PGWC). These traits showed extensive genetic variation and varied according to MAGIC populations (DC1, DC2 and 8way) with different GBs, suggesting that phenotypic variation largely depended on the parents and their recombination in MAGIC populations. Rice grain type has high heritability and was mainly affected by genotype (Zhao et al., 2022). However, chalkiness was caused by the deposition of starch and storage proteins in the endosperm, which was controlled by a complex genetic mechanism and sensitive to environment (Ishimaru et al., 2020; Liu et al., 2022). In this study, grain shape (GL, GW and GLWR) showed consistent performances across the four environments (15_PX, 15_SZ, 16_PX and 16_SZ), while appearance quality traits (DEC and PGWC) showed great differences according to environments. These results were consistent with previous studies (Ishimaru et al., 2020; Liu et al., 2022; Zhao et al., 2022).

Based on correlation analysis between different traits, DEC and PGWC showed consistently significantly positive correlations with GW but no significant correlation with GL (**Figure 2**), suggesting that there was no inevitable effect of GL on chalkiness (Zhao et al., 2022). QTL mapping for grain shape-related traits or chalkiness was often delimited to the same QTL cluster (Gong et al., 2017), suggesting that there might be a close relationship between grain shape and chalkiness (Zhao et al., 2016). In our study, *AqGW2* and *AqGW5a* for GW were mapped in the same regions with *AqDEC2* for DEC in the region of 31.39−32.20 Mb on chromosome 2, and *AqDEC5* for DEC and *AqPGWC5* for PGWC5 in the region of 5.16−5.56 Mb on chromosome 5, respectively, while there was no any QTL for GL mapped together with that for DEC and PGWC. These results suggested that chalkiness could be ameliorated by regulating GW.

### Comparisons of QTL detected in this study with previously reported cloned genes

Among the 22 QTL associated with the five grain appearance quality-related traits detected by GWAS, 10 QTL covered or were adjacent to previously reported cloned genes in rice (**Supplementary Table S3**). For example, *AqGL3b* for GL and *AqGLWR3a* for GLWR, which was detected in the region of 16.47–17.24 Mb on chromosome 3 in all seven populations except for 8way, were mapped together with the previously reported gene *GS3* for grain length and weight (Mao et al., 2010). *AqDEC6b* influencing DEC in the region 1.50–2.19 Mb and *AqPGWC6b* influencing PGWC in the region 1.54–2.19 Mb on chromosome 6 were co-located with *GS6*, a gene increasing grain width and weight significantly by a premature stop at the t348 position in the coding sequence (CDS) (Sun et al., 2013). The QTL cluster in the region of 5.16−5.62 Mb on chromosome 5, including *AqGW5a*, *AqGLWR5*, *AqDEC5* and *AqPGWC5*, was co-located with the previously reported gene *GW5*, which significantly affected grain width and weight of rice (Liu et al., 2017). *AqGW2* for GW and *AqDEC2* for DEC mapped in the region of 31.39−32.20 Mb on chromosome 2 in DC128 and DC1, respectively, were co-located with *qTGW2* regulating grain width and weight by natural variation in the promoter (Ruan et al., 2020).

Among the 43 QTL associated with the five grain appearance quality-related traits detected by LA, eight QTL covered or were adjacent to previously reported cloned genes in rice (**Supplementary Table S4**). *GqGL3a* for GL and *GqGLWR3b* for GLWR detected in the region of 14.22−17.94 Mb on chromosomes 3 were co-located with *GS3* (Mao et al., 2010). *GqGW5a*, *GqGLWR5*, *GqDEC5a* and *GqPGWC5a* for multiple grain shape and appearance traits detected in the region of 0.59−1.68 Mb on chromosome 5 were mapped together with the previously reported gene *GW5* (Liu et al., 2017). *GqDEC6* for DEC and *GqPGWC6* for PGWC detected in 0.59−1.69 Mb on chromosome 6 were co-located with the previously cloned gene *GS6* (Sun et al., 2013). Allelic correspondences of the above QTL influencing the grain appearance quality-related traits identified in this study with previously reported genes will need to be further verified by fine mapping and QTL cloning.

### Influence of GB and population size on QTL mapping

GB had strong effect on QTL mapping of complex quantitative traits (Moncada et al., 2001; Septiningsih et al., 2003; Xie et al., 2006), even in reciprocal introgression line populations which were derived from two parents and shared partial common genome (Mei et al., 2006; Cheng et al., 2012; Wang et al., 2013). In this study, different common QTL were detected by GWAS and LA in DC1 and 8way, DC2 and 8way, and DC1, DC2 and 8way. There was also no any QTL simultaneously detected by the two mapping approaches both in DC1 and DC2. The GB of DC1 and DC2 were completely different but partially the same as that of 8way. These results further confirmed that GB largely affect the mapping results, and it seems easier to map the common QTL in populations with similar GB.

Total of 12, eight and 14 QTL were averagely identified for the five grain-shape and appearance traits by GWAS in DC1, DC2 and 8way, respectively. It was indicated that eight-way population is more powerful than two four-way populations, consistent with the expectation that progeny generated from multiple parents offers an advantage in that more QTL can be detected. However, there were QTL that were not detected using the 8-way population were detected using the DC1 (i.e., *AqDEC2* and *AqDEC6c*), DC2 (i.e., *AqGL3a* and *AqPGWC8*). When only the QTL corresponding to known genes were considered, the QTL undetected by the 8-way population is the *AqDEC2* co-located with reported gene *qTGW2* for the DC1 population. The more parents involved in developing a population, the lower frequencies of different alleles are usually presented, thus resulting in reduced power for detecting QTL with small effects.

Combining different single population to expand the population size for mapping QTL by GWAS, we found that the number of QTL for grain appearance quality mapped in the single population was comparable to that in the merged population. DC12 population was derived from different parents allows detecting QTL that are segregating only in one of the populations and as a result are not detectable in the other population. Furthermore, when a single population reaches a certain size (~400), continuing to expand the population size by combining different populations such as DC18, DC28 and DC128, which share some partial common parents, didn’t also necessarily improve the effectiveness of mapping results. Obviously, if all the parents were used to develop a single population, much higher power for QTL mapping would be achieved.

### QTL mapping efficacy of the two mapping methods

Total of 12, 8 and 14, and 16, 19 and 16 QTL for the five traits were identified by GWAS and LA in DC1, DC2 and 8way, respectively. Clearly, the number of QTL detected by LA was more than that detected by GWAS. The background (noise) control adopted in ICIM can effectively reduce the sampling variance by controlling the effects of QTL located on other intervals and chromosomes, thus will increase the power of QTL mapping (Zhang et al., 2019). Among them, 8, 3 and 7 QTL were simultaneously detected by GWAS and LA in the same regions in DC1, DC2 and 8way, respectively. The average phenotypic variance explained by above commonly detected QTL was 11.03%, significantly higher than that of other QTL with the average PVE 4.84%, ranging from 0.57% for *GqDEC1* in 15_PX in DC2 to 15.31% for *GqGLWR* in 16_PX in DC2. These results indicated that GWAS mainly identified QTL with relatively large effect, while LA could identify more QTL with small effect in addition to QTL with large effect.

At present, most genes cloned by forward genetics were initially mapped by LA, and then further fine-mapped by identifying more recombinants in the target region. This method had the advantage of high localization accuracy. GWAS is based on linkage disequilibrium, and uses single nucleotide polymorphisms (SNP) in the genome as molecular genetic markers to conduct correlation analysis at the genome-wide level to map genes. It can detect multiple alleles at the same locus simultaneously. Population genetic structure and allele frequencies could lead to false-positive association results. In addition, SNP data might be filtered out when association analysis was performed for some rare alleles which probably affect important phenotypes (Tao et al., 2018). Furthermore, association analysis could only map large interval around significant SNPs according to the LD decay distance. Considering characteristics of the two mapping methods, joint mapping of QTL by GWAS and LA can identify more reliable QTL. To date, great progress had been made in rice gene cloning using a combination of GWAS and LA, such as *OsSPL13* (Si et al., 2016), *bsr-d1* (Li et al., 2017), and *RH8* (Li and Li, 2016).

### Candidate gene identification for important QTL

Through gene-based association analysis and haplotype analysis, 14 candidate genes were identified for newly identified QTL, *AqGL3c/AqGLWR3b*, *AqDEC6a/AqPGWC6a*, and *AqGL7* affecting grain appearance quality-related traits. The most likely candidate genes were further inferred by gene annotation and the expression pattern of candidate genes for each QTL using the RNA-seq database from MBKBASE.

GL is coordinately regulated by cell expansion and cell proliferation that involve in multiple signaling pathways and biological processes (Li et al., 2018). Among four candidate genes for *AqGL3c/AqGLWR3b*, *Os03g0586700*, highly expressed in milk and mature stage of rice grain, encodes a protein tyrosine phosphatase-like protein, which participated in the regulation of cell proliferation and differentiation (Bellec et al., 2002) (**Supplementary Figure S1A**), suggesting that *Os03g0586700* is the most likely candidate gene and worthy of further verification by gene-editing or transgenic approach.

Among the four genes of *AqDEC6a* and *AqPGWC6a*, *Os06g0102300* (*OsPCS2*) encodes chelating peptide synthase and has two variably cleaved transcripts, *OsPCS2a* and *OsPCS2b*, which has unconventional premature stop codons (Das et al., 2017). *OsPCS2a* and *OsPCS2b* were expressed in roots, stems and developing seeds (**Supplementary Figure S1B**). *Os06g0111800* was expressed in grain endosperm and encodes a cellulose synthase-like D protein, which was involved in cell wall and vascular bundle formation that influences photosynthesis (Yin et al., 2011; Flexas et al., 2021) (**Supplementary Figure S1B**). Therefore, *Os06g0111800* and *Os06g0102300* are inferred as most likely candidate genes affecting DEC and PGWC.

Among eight candidate genes for *AqGL7*, *Os07g0666600* encodes a kinase interacting family protein. The orthologous of *Os07g0666600* in Arabidopsis is cytoskeleton proteins NETWORKED 3A/C that functions as cell morphology modulators as well as scaffolds to recruit other components, and ultimately regulates cell size (Hussey et al., 2006; Wang et al., 2014) (**Supplementary Figure S1C**). The degree of chalkiness is highly related to the “source-sink” in rice. So, *Os07g0666600* is inferred as a most likely candidate gene affecting GL.

For the candidate gene *Os03g0586700*, Hap2 was the dominant haplotype with higher GL and GLWR (**Figure 4**). Haplotype analysis of *Os07g0666600* revealed that Hap2 was associated with significantly larger GL than Hap1 both in SZ and SY (**Figure 5**). Hap4 of *Os06g0102300* had lower DEC and PGWC both in SZ and SY, suggesting that Hap4 was the dominant haplotype to improve chalky quality in rice (**Figure 6**). For *Os06g0111800*, Hap3 was the dominant haplotype to improve chalky quality in rice (**Figure 6**). These dominant haplotypes could be used in molecular breeding of rice to improve the grain appearance quality of rice.

### Implication for breeding of high appearance grain quality

Through comprehensive comparisons of QTL detected by GWAS and LA in this study, seven important QTL including *AqGL3b*/*AqGLWR3a* (*GS3*), *AqGL3c*/*AqGLWR3b, AqGW5a*/*AqGLWR5*/*AqDEC5*/*AqPGWC5* (*GW5*), *AqDEC6a*/*AqPGWC6a*, *AqDEC6b*/*AqPGWC6b* (*GS6*), *AqGL7* and *AqPGWC7* were stably detected for grain appearance quality-related traits across four environments. GW was found to positively correlate with chalkiness traits (DEC and PGWC) in this study and in the previous studies (Li et al., 2014; Qiu et al., 2015; Zhao et al., 2015; Zhou et al., 2015). So, QTL cluster of *AqGW5a*/*AqGLWR5*/*AqDEC5*/*AqPGWC5* affecting both grain shape (GW and GLWR) and chalkiness traits (DEC and PGWC) could be used for improvement of appearance quality by reducing GW or enlarging GLWR. In general, slender grain with large GLWR tends to have good grain appearance quality (McKenzie and Rutger, 1983). High grain appearance quality could be also realized by combining *AqGL3c*/*AqGLWR3b* affecting GLWR and GL with *AqDEC6a*/*AqPGWC6a* or *AqDEC6b*/*AqPGWC6b* for DEC and PGWC to get a perfect combination of grain type and appearance quality.

In the present study, two groups of lines were selected to have good performance of GLWR and DEC/PGWC. Group 1 including L1352, L1422 and L1692 were selected based on largest GLWR (4.18) and relatively low DEC (3.58%−4.74%)/PGWC (11.74%−17.33%) from the three single MAGIC populations, and group 2 including L1660, L1716 and L1745 were selected based on lowest DEC (2.10%−2.73%)/PGWC (9.29%−9.35%) and relatively large GLWR (3.54−3.96) (**Supplementary Table S2**). The selected lines have different patterns of favourable alleles at all seven important QTL except *AqGL3b*/*AqGLWR3a* and *AqDEC6a*/*AqPGWC6a* for grain appearance quality-related traits. We can further improve appearance quality by pyramiding non-allelic favourable alleles distributing on different chromosomes. For example, DEC and PGWC can be further improved by making L1352 or L1442 crossed with L1660 or L1671 to pyramid three favourable alleles at *AqDEC6b*/*AqPGWC6b*, *AqGL7* and *AqPGWC7*, or making L1352 or L1442 crossed with L1745 to pyramid two favourable alleles at *AqGL7* and *AqPGWC7* by following QTL designed pyramiding strategy. Of course, these eight lines could be directly used as donor parents to improve grain appearance quality of elite rice variety with poor appearance quality by marker-assisted selection.

## Conclusions

A total of 22 QTL were identified for five traits in DC1, DC2 and 8way, and four combined populations (DC12, DC18, DC28 and DC128) by GWAS, and 42 QTL were identified in the three single populations by LA. GB had strong effect on QTL mapping both for GWAS and LA. The 8way population was more powerful for QTL mapping than the DC1, DC2 and various combined populations. Compared with GWAS, LA can identify more QTL with minor-effect besides large-effect QTL. Among 11 QTL simultaneously detected by the two methods, eight QTL corresponded to known genes, and four QTL (*AqGL7*, *AqGL3c/AqGLWR3b*, *AqDEC6a/AqPGWC6a*, and *AqPGWC7*) were considered as novel QTL and their candidate genes were inferred. Based on distribution of non-allelic favourable alleles for appearance quality among promising lines in the three MAGIC populations, it is feasible to further enhance appearance quality by pyramiding different favourable alleles using QTL-designed-pyramiding strategy.

## Data availability statement

The original contributions presented in the study are included in the article/**Supplementary Material**. Further inquiries can be directed to the corresponding authors.

## Author contributions

HC, KC, CS, SZ, JT and JL performed the experiments. LZ and HC drafted the manuscript. LZ and PQ did data analysis. JX and HH designed the experiments and revised the manuscript. All authors contributed to the article and approved the submitted version.

## Funding

This work was founded by the Key Research and Development Project of Hainan Province (ZDYF2021XDNY128), the Agricultural Science and Technology Innovation Program and the Cooperation and Innovation Mission (CAAS-ZDXT202001), and Supported by the earmarked fund for China Agriculture Research System (CARS)-01.

## Acknowledgments

We gratefully thank Drs Guoyou Ye and Xiangqian Zhao for providing the seeds of MAGIC populations.

## Conflict of interest

The authors declare that the research was conducted in the absence of any commercial or financial relationships that could be construed as a potential conflict of interest.

## Publisher’s note

All claims expressed in this article are solely those of the authors and do not necessarily represent those of their affiliated organizations, or those of the publisher, the editors and the reviewers. Any product that may be evaluated in this article, or claim that may be made by its manufacturer, is not guaranteed or endorsed by the publisher.
