# Supplementary figures and images for: Genetic background- and environment-independent QTL and candidate gene identification of appearance quality in three MAGIC populations of rice
Source: Front Plant Sci. 2022 Nov 11;13:1074106. doi: 10.3389/fpls.2022.1074106 (PMC9697191; doi:10.3389/fpls.2022.1074106)

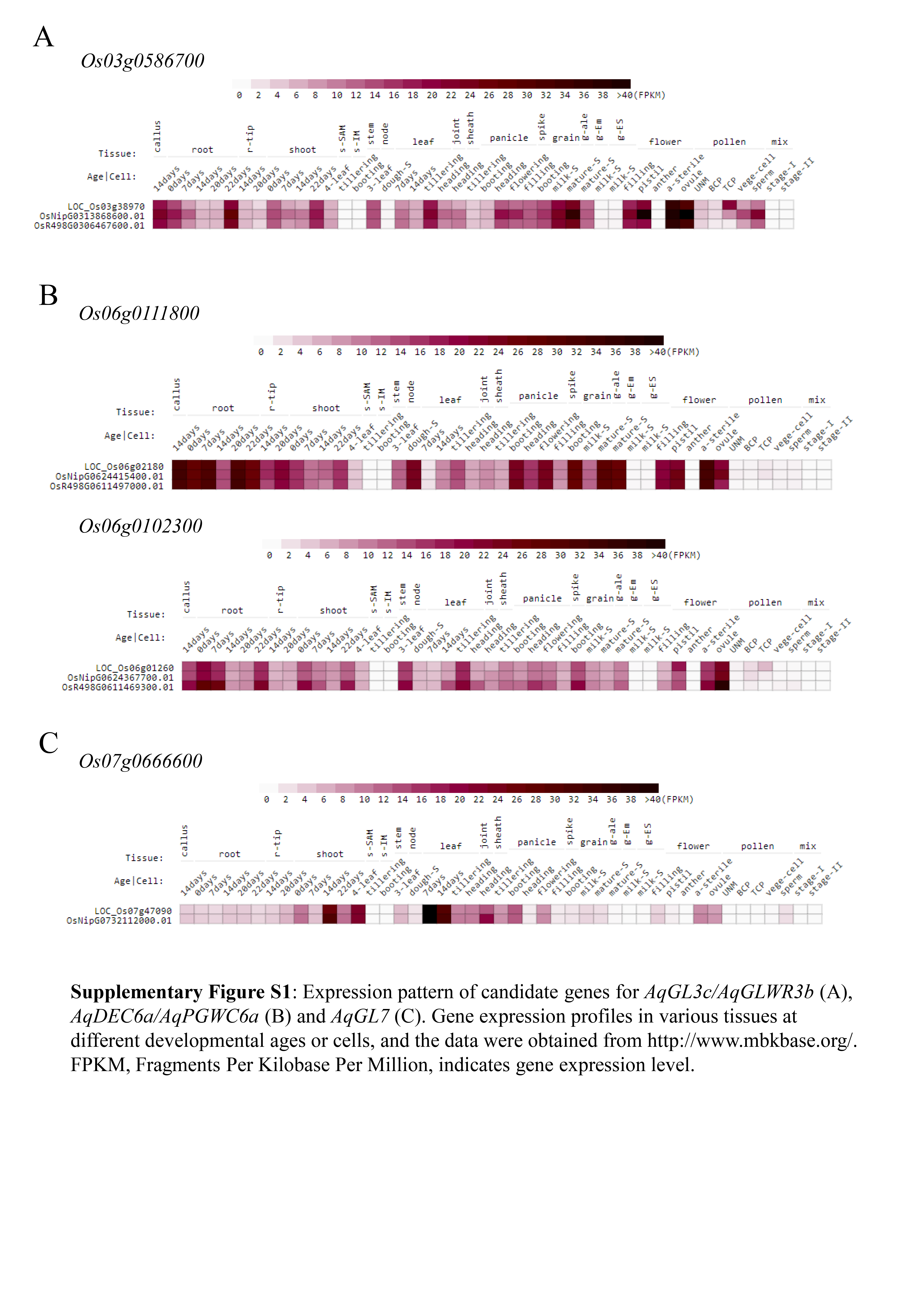

Supplement: Supplementary file 1 [file Image_1.tif]
